# Supplementary material for: Patterns of Intron Gain and Loss in Fungi
Source: PLoS Biol. 2004 Nov 30;2(12):e422. doi: 10.1371/journal.pbio.0020422 (PMC532390; doi:10.1371/journal.pbio.0020422)
Supplement: Table S1 — Also available at http://genes.mit.edu/NielsenEtAl/. (4.3 MB ZIP). [file pbio.0020422.st001.zip › NielsenEtAl/html/1259.html]

AN0736.1.NCU04122.1.MG09188.1.FG01872.1


```
 CLUSTAL W (1.82) Multiple Sequence Alignments - Introns Inserted


Sequence 1: NCU04122.1	270 aa
Sequence 2: MG09188.1	263 aa
Sequence 3: FG01872.1	264 aa
Sequence 4: AN0736.1	247 aa
Alignment Length: 291 aa
Number Identitical Residues: 68 aa
Alignment Score (without introns) 3610


MG09188.1 	MYSSIILATAFIASAFAAP--ANPNLNLASAHPGSHSAVSDYFNMLAKKVQEAKQLPQGG
NCU04122.1	MRASLFLSALSVSSIMAAP--LLPEINAHSAPADSLKKISEYFNLLAVKIQQSK-FESAV
FG01872.1 	MQLSTFVASVLAVTASAAPSFPKPTITDTRSAIDTLGSLSGYFNLVAEKVKAAKAYDVA-
AN0736.1  	-MRSVLLISCLAACSAAAP------------IFENFYNFSDDMAEFLGRVSKAIHSEVNS
          	   * :: :       ***               .   .*  :  .  ::. :       

MG09188.1 	PVCDLSSVQLPTV~PGS1KEPMPLPGAGYKLKHVALGRGTQ~NYTCSPNNATAIPAAAGA
NCU04122.1	PTCDLSKVVMPQA1P--~-SPLPPPSFGLTLRHVALGRGTQ~NYTCDPSTPTAAPVANGA
FG01872.1 	PVCDLSQAKMALD1G--~--QLLPPSKGLYLKHVAIGRGTQ~NYTCDTKNPSSAPVATGA
AN0736.1  	LTCDTSSIALPSF~AS-~--GLPAPT-GQKPLYVALGRGTQ0NYTCADSTSSSTPEAVGA
          	 .** *.  :.    .     :  *  *    :**:***** ****  ...:: * * **

MG09188.1 	VATLFDASCIASAYPALLMELSRVTVNFNLTDLN-------------------TLAPSN-
NCU04122.1	VASLFNASCIVSAYPDIGAMLSTVSLNFNLSDLASIASSVASALPFPPSFLKQTLAPTSG
FG01872.1 	VATLFNASCAAALYPDLVERIPGMAVHFPLTDAA-------------------KLGPAS-
AN0736.1  	VARLYNATCLAARLPKAIELLPAIAYKLTLPANEY-----------------DPLPPSN-
          	** *::*:* .:  *     :. :: :: *.                     . * *:. 

MG09188.1 	LLMSGHHYFAPNITVPYFDLGP-----LGSCMAAKNASEPAPADAGKGQGGEPAVPWLKL
NCU04122.1	MAVSGAHYFT-NASTPFFNMDAS-QWKIGEAPCAKNSSTPAPEAAPRGQQGEKAVAWLKL
FG01872.1 	FPESGHHYFT-ADGVPFFDLRTP-EQTIGEAPCAKNSSAPAPSFSAKGQLGEAAVPWLRL
AN0736.1  	FNLLGHHFFE--GKVPVFDLDTTPDRQLGIAKVKKEADLPAPTSAVRGDDDEGAVAWLYL
          	:   * *:*     .* *:: ....  :* .   *::. ***  : :*: .* **.** *

MG09188.1 	TTREGATGDLQEVYRVGTAGGSAPATCKDMPATFEVQYTAQ2YWFYAGKKPTQE-
NCU04122.1	VTRPGATGGLQEVYRVETAGGSAPETCQGMPEHFEVQYAAQ~-------------
FG01872.1 	FTIKGATHDIKEVYRTTTAGGSAPATCNGMASEFEVQYATL2YWFWAGKTDDAEA
AN0736.1  	SRSNGTVGRYSSVYRVDTAGGSPPETCEGMARSFEVEYAAN~YYIYGQ-------
          	    *:.   ..***. *****.* **:.*.  ***:*::      .
```
